# Catalyst accessibility to chemical reductants in metal–organic frameworks†
†Electronic supplementary information (ESI) available: Experimental details, preparation and characterization of MOFs, additional spectra of the reduced MOF. See DOI: 10.1039/c7cc00022g


**DOI:** 10.1039/c7cc00022g

**Published:** 2017-02-23

**Authors:** Souvik Roy, Vlad Pascanu, Sonja Pullen, Greco González Miera, Belén Martín-Matute, Sascha Ott

**Affiliations:** a Uppsala University , Department of Chemistry – Ångström Laboratory , Box 523 , 751 20 Uppsala , Sweden . Email: sascha.ott@kemi.uu.se; b Department of Organic Chemistry , Arrhenius Laboratory , and Berzelii Center EXSELENT , Stockholm University , 10691 Stockholm , Sweden . Email: belen.martin.matute@su.se

## Abstract

A molecular H_2_-evolving catalyst, [Fe_2_(cbdt)(CO)_6_] ([FeFe], cbdt = 3-carboxybenzene-1,2-dithiolate), has been attached covalently to an amino-functionalized MIL-101(Cr) through an amide bond. Chemical reduction experiments reveal that the MOF channels can be clogged by ion pairs that are formed between the oxidized reductant and the reduced catalyst. This effect is lessened in MIL-101-NH-[FeFe] with lower [FeFe] loadings. On longer timescales, it is shown that large proportions of the [FeFe] catalysts within the MOF engage in photochemical hydrogen production and the amount of produced hydrogen is proportional to the catalyst loading.

## Abstract 2

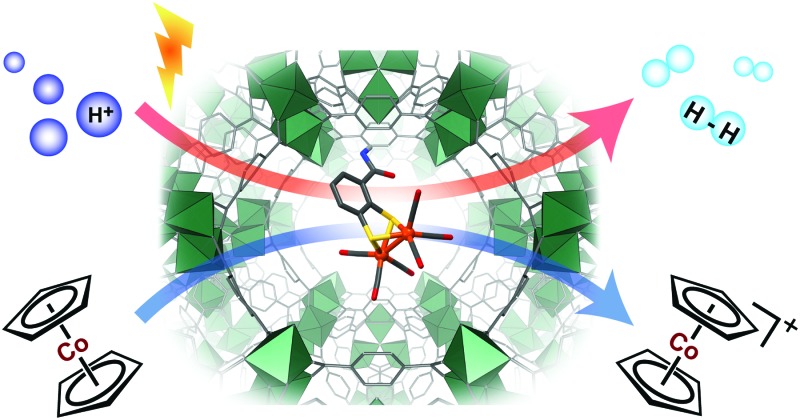
This study of catalyst accessibility inside metal–organic frameworks demonstrates that pore dimensions, catalyst loadings, concentration of reductant, and reaction times all influence the proportion of catalysts within MOFs that engage in redox chemistry.

## 

Efficient catalysts for the (photo)electrochemical hydrogen evolution reaction (HER) are central to future carbon-free energy systems.^1^–^3^ One of the more recent strategies has focused on metal–organic frameworks (MOFs), *i.e.* large surface area mesoporous coordination polymers, as matrices for HER catalysts.^4^–^6^ Heterogeneous nanoparticles and polyoxometalates have been incorporated into MOFs and the resulting materials used for the HER.^7^–^12^ The molecular nature of the MOF however also permits the controlled introduction of molecular catalysts, either by solvothermal synthesis or by post-synthetic methods.^13^ The resulting catalyst-containing MOFs (hereafter termed MOFcats) are motivated for mainly two reasons: catalyst immobilization will most likely lead to improved structural stability of the catalyst and thus higher turnover numbers, while the remaining organic linkers in the MOF can carry further functional groups that can interact with the incorporated catalysts, thereby offering a tailor-made active site cavity that would be reminiscent of the situation in enzymes.^14^,^15^ Realization of this long-term vision requires a detailed mechanistic understanding of how MOF incorporation influences the catalysts’ modes of action. With a few exceptions, such spectroscopic studies of catalyst intermediates in MOFcats are at present largely absent in the literature.^16^ Taking the analogy to enzymes one step further, it is also clear that access channels to the active sites are crucial for activity. This is particularly important for MOFcats which are driven by (photo)chemical reductants as these species have to diffuse hundreds of nm to access the catalyst sites that are deeply buried inside the MOFcat crystals. Along those lines, Hupp, Farha, Kubiak and co-workers have recently reported a MOF as a “substrate delivery system” that very efficiently shuttles protons to a heterogeneous Ni–S electrocatalyst, thereby decreasing the kinetic overpotential by 200 mV.^17^

For molecular catalysts incorporated inside MOFs, systematic studies that address catalyst accessibility are to the best of our knowledge absent in the literature. Herein, we present for the first time a comparative study that shines light on the accessibility of catalysts when incorporated inside different MOFs. At the same time, a reduced intermediate of the catalytic cycle is spectroscopically characterized, illustrating how MOF incorporation can prevent catalyst deactivation pathways. The catalyst of choice is [Fe_2_(c_*x*_bdt)(CO)_6_] ([FeFe]) in which the benzene-1,2-dithiolate (bdt) ligand carries carboxylic acid groups (*x* = 1 or 2) that allow MOF incorporation. An advantage of this catalyst is that it contains CO ligands that are invaluable IR probes that reveal the oxidation state of the catalyst.^18^ MIL-101(Cr)^19^ and UiO-66(Zr)^20^ were chosen for this study due to their extraordinary stability in aqueous solution at low-pH.^21^ MIL-101 exhibits larger pore diameters (29–34 Å), compared to those of UiO-66 (8–11 Å), and also provides easier access to these pores through windows of 12–14.5 Å compared to 6 Å in UiO-66.^22^,^23^ [Fe_2_(c_1_bdt)(CO)_6_]^24^ was covalently incorporated into an amino-functionalized MIL-101(Cr)-NH_2_ by reacting the latter with a mixed-anhydride precursor of [Fe_2_(c_1_bdt)(CO)_6_] (Scheme S1, ESI†). By adjusting the reaction time and concentration of the precursor solution (see the ESI† for details), a series of MIL-101-NH-[FeFe] materials with low, medium and high catalyst loadings of 0.59 (low), 2.35 (medium) and 5.28 (high) wt% Fe, respectively, could be synthesized; a loading that corresponds to 2.2%, 9.4% and 24.3% of all linkers being functionalized, respectively. Thus, for the MIL-101-NH-[FeFe]-high, approximately one of four linkers carries an [FeFe] unit. An inevitable consequence of such high loading is that catalysts must be present throughout the entire material, and cannot be localized only on the surface of the MOF crystals. IR and XAS data (Fig. 1 and Fig. S1, ESI†) confirm the structural integrity of the [FeFe] complex after MOF incorporation, while PXRD and SEM verify that the crystallinity of the material is maintained (Fig. S2 and S3, ESI†). The material was further characterized by thermogravimetric analysis (TGA) and BET surface area measurements which confirm the incorporation of the complex inside MIL-101 (Fig. S4–S6, ESI†). UiO-66-[FeFe] was prepared by the incorporation of [Fe_2_(c_2_bdt)(CO)_6_] into UiO-66 using a post-synthetic exchange (PSE) protocol which results in 14% of the organic linkers being exchanged by the [FeFe] catalyst.^24^

**Fig. 1:**
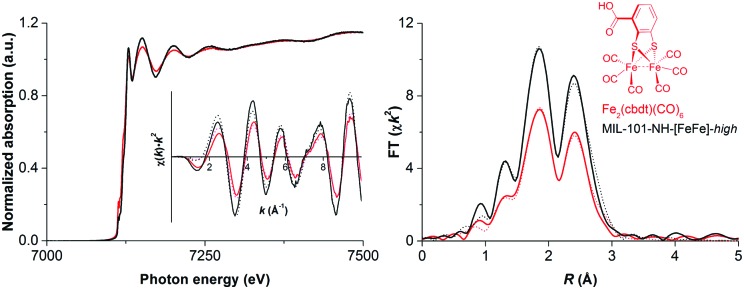
Fe K-edge XAS spectra (left) with *k*-weighted EXAFS functions *χ*(*k*)*k*^2^ (inset) and Fourier transforms of EXAFS functions in R space (right) for the molecular catalyst (Fe_2_(cbdt)(CO)_6_, red) and the MOF-material (MIL-101-NH-[FeFe]-high, black). Solid lines show experimental data and dashed lines show fits from crystallographic data.

First indications of the effect that different pore and window sizes have on catalyst accessibility were obtained in chemical reduction experiments, using the CO ligands at [FeFe] as IR probes to follow electron transfer. Cobaltocene (Cp_2_Co) was chosen as the reducing agent on grounds of its size (*ca.* 6.6 Å × 6.6 Å, corrected for van der Waals radii) and reduction potential (*E*_1/2_ = –1.35 V *vs.* Fc^+/0^ in CH_3_CN).^25^,^26^ Assuming a reduction potential of the [FeFe] complex in the MOFs that is similar to that of [Fe_2_(c_*x*_bdt)(CO)_6_] (*x* = 1, 2) in the homogenous phase,^27^ a driving force for an electron transfer of *ca.* 150 mV can be estimated.

As shown in Fig. 2, addition of five equivalents of Cp_2_Co (with respect to [FeFe]-catalyst) to a suspension of MIL-101-NH-[FeFe]-high in CH_3_CN leads to the consumption of *ca.* 36% of the starting material and the emergence of new IR absorptions at 1882, 1928, and 1978 cm^–1^. This observation is vastly different to that of the analogous reaction with UiO-66-[FeFe] where the addition of similar quantities of Cp_2_Co has little effect on the IR spectrum (Fig. S7, ESI†). This finding could in parts be explained by a sheer size argument, as Cp_2_Co accessibility into the interior of the UiO-66 crystals could be limited by the pore windows which are similar in dimension. An alternative explanation is that small portions of the [FeFe] sites at the periphery of the crystals are getting reduced and form stable ion pairs with oxidized [Cp_2_Co]^+^. Such an ion pairing would cause pore clogging and prevent access to catalysts that are more deeply buried in the UiO-66 crystal.

**Fig. 2:**
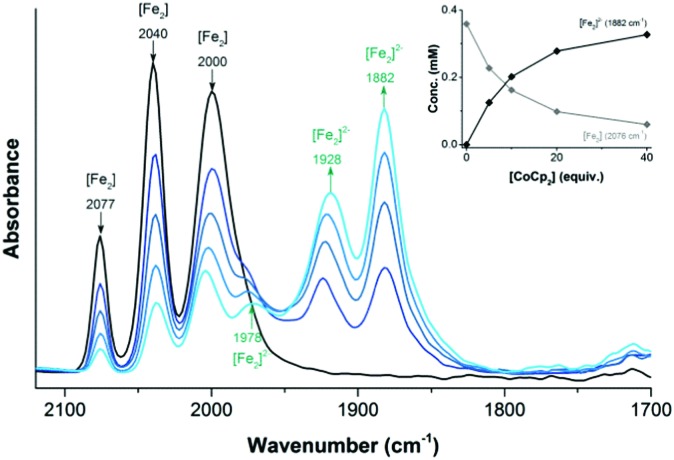
FT-IR monitoring of MIL-101-NH-[FeFe]-high (black) reduction with CoCp_2_: spectra recorded 10 min after addition of CoCp_2_ (black to pale blue: 0, 5, 10, 20 and 40 equiv. CoCp_2_). Inset shows the change in concentration of [FeFe] and [FeFe]^2–^ with increasing amount of CoCp_2_.

The IR spectrum of the reduction product of MIL-101-NH-[FeFe]-high is very similar to that of the doubly reduced species [FeFe]^2–^ which is observed as the product of electrochemical reduction due to the inverted electrochemical potential of [FeFe] and the [FeFe]^–^ monoanion.^3^,^14^,^28^ The *ν*_C–O_ bands of the [FeFe]^2–^ species inside the MIL-101 are however slightly higher in energy (∼13–17 cm^–1^) compared to those of its solution-phase bdt-analogue (Fig. S8, ESI†). We assign this difference to the formation of stable ion-pairs between the oxidized [Cp_2_Co]^+^ and the reduced [FeFe]^2–^ inside the MOF-cavities (*vide supra*). Increasing the amount of added Cp_2_Co leads to the reduction of additional [FeFe] sites (Table S4, ESI†), and a 40-fold excess relative to the [FeFe] content in MIL-101-NH-[FeFe]-high (pale blue trace, Fig. 2) leads to a final state where *ca.* 82% of all [FeFe] sites within the MIL-101 have been reduced. The lack of complete transformation despite of the large excess of CoCp_2_ points towards a clogging of the framework channels and a thereby caused inaccessibility of some of the catalysts. If this hypothesis is correct, a decrease in catalyst loading should lessen this limitation. Indeed, identical reduction experiments with MIL-101-NH-[FeFe]-medium proved to proceed with higher yields (Table S4 and Fig. S9, ESI†), and *ca.* 90% of all catalysts could be chemically reduced. Quantification of the reduced complex in MIL-101-NH-[FeFe]-low is limited by a poor signal-to-noise ratio, but the IR spectrum shows almost quantitative reduction (Fig. S10, ESI†). It is thus interesting to note that catalytic MOF materials of one and the same MOF and structurally identical catalytic sites may show different substrate accessibility, purely as a function of catalyst loading.^29^

With strong indications for the presence of ion pairs, focus was turned on to their stability over time. Addition of 10 equiv. of Cp_2_Co to MIL-101-NH-[FeFe]-high results in the formation of about 40% [FeFe]^2–^ already after five minutes, and 55% after 10 minutes (Fig. 3). After this initial phase, the reaction becomes more sluggish, but continues for at least four hours when 61% of the MIL-101-bound [FeFe] complex is converted to [FeFe]^2–^ (green trace, Fig. 3). In addition to the gradual consumption of [FeFe], a small shift of 13 cm^–1^ can be observed in the product absorption at 1928 cm^–1^ on these timescales. As the overall spectral shape does not change, we attribute this shift either to small structural changes in [FeFe]^2–^ or, more likely, to the dissociation of ion pairs. The latter explanation is consistent with the liberation of oxidized [Cp_2_Co]^+^ from the material, providing access to additional [FeFe] sites that can be reduced to [FeFe]^2–^ over these long timescales. Importantly, PXRD analysis of cobaltocene-treated MIL-101-NH-[FeFe]-high confirms that the MOF framework remains intact after reduction of catalyst units by Cp_2_Co (Fig. S11, ESI†).

**Fig. 3:**
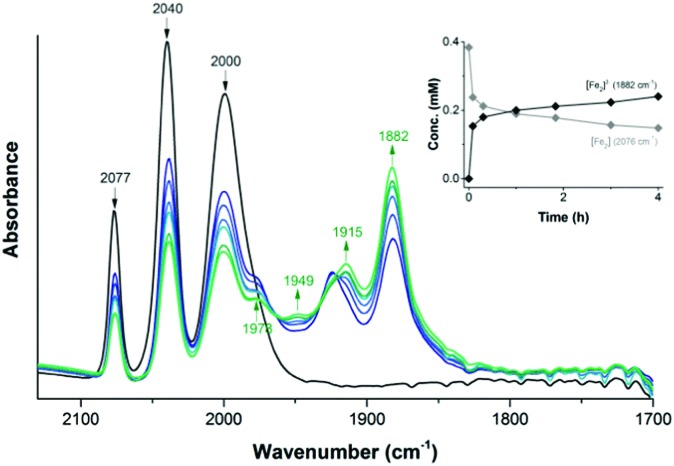
FT-IR monitoring of MIL-101-NH-[FeFe]-high (black) reduction with CoCp_2_: time-dependent spectral changes in the presence of 10 equiv. of CoCp_2_ (navy blue to green: 0, 5, 18, 50, 110, 180 and 240 min.). Inset shows change in conc. of [Fe_2_] and [Fe_2_]^2–^ over time.

The timescale at which the [FeFe]^2–^ remains structurally intact is remarkable. While the reduced catalyst state [FeFe]^2–^ is stable for hours in MIL-101, the same species has been shown to form dimers and higher nuclearity clusters in homogenous phase within minutes (Fig. S12, ESI†).^30^–^33^ The experiments thus illustrate very impressively the capacity of the MIL-101 matrix to stabilize reactive intermediates by keeping them structurally isolated.

Having proven that large proportions of the catalysts are accessible in MIL-101-NH-[FeFe], the performance of the three materials in the photocatalytic HER was evaluated. In a typical experiment, the three MIL-101-NH-[FeFe] samples were separately suspended in a solution of ascorbic acid (0.1 M) and [Ru(bpy)_3_]^2+^ (0.5 mM) in acetate buffer (1.0 M, pH = 4.9), and illuminated with 470 nm light. Hydrogen evolution was monitored by gas chromatography. As shown in Fig. 4, all three MIL-101-NH-[FeFe] materials are active photocatalysts and, importantly, display better catalytic activity compared to the homogenous reference system with [Fe_2_(dcbdt)(CO)_6_] (Fig. S13, ESI†). Hydrogen evolution was not observed in the absence of light.

**Fig. 4:**
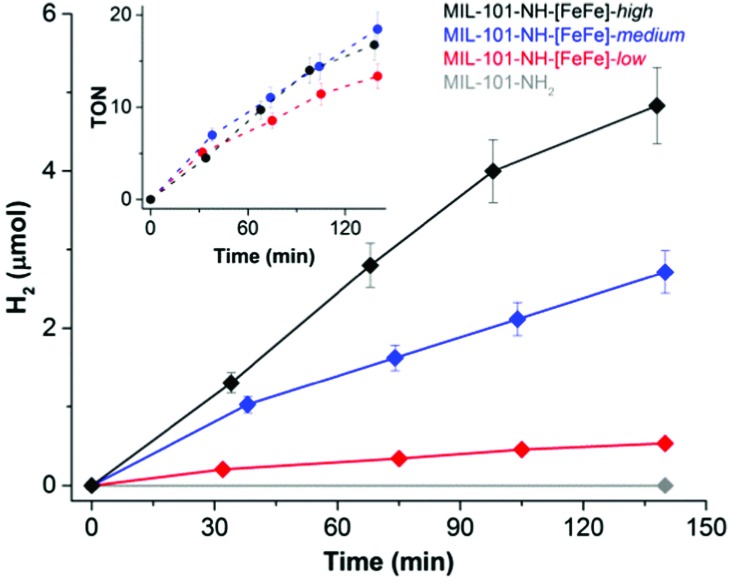
Photocatalytic H_2_ evolution in the presence of MIL-101-NH-[FeFe]-high (black, ∼0.29 μmol cat.), MIL-101-NH-[FeFe]-medium (blue, ∼0.15 μmol cat.), MIL-101-NH-[FeFe]-low (red, ∼0.04 μmol cat.) and MIL-101-NH_2_ (grey, 0 μmol cat.). Inset: catalytic turnover numbers (TON) for H_2_ evolution (TON = μmoles of H_2_ per μmol of catalyst) based on total catalyst loading. Reaction conditions: 1.0 M acetate buffer pH 4.9, 0.1 M ascorbic acid, 0.5 mM [Ru(bpy)_3_]^2+^, blue LED (470 nm).

When comparing hydrogen evolution from MIL-101-NH-[FeFe]-high to that of MIL-101-NH-[FeFe]-medium (Fig. 4, and Fig. S14, ESI†), it turns out that an increase in the catalyst loading by a factor of 2.5 leads to a proportional increase in hydrogen production within experimental error. Similarly, a fourfold increase in the catalyst concentration from MIL-101-NH-[FeFe]-low to MIL-101-NH-[FeFe]-medium leads to a fourfold increase in the amount of evolved hydrogen (Fig. 4). Consequently, the catalytic turnover numbers (TONs = mol H_2_ per mol [FeFe]) are very similar for the three materials (Fig. 4, inset). Hydrogen production is thus linearly dependent on the catalyst loading in the MOF, and catalysis is first order in catalyst (Fig. S15, ESI†). The results are consistent with catalysis not being limited by the photoproduction of the reductant as is often the case in photochemical systems, but by the intrinsic turnover rate of the catalyst within its environment.^34^–^36^

The TON calculation requires the assumption that all catalysts throughout the MIL-101 engage in catalysis. While this scenario is not necessarily the case, it can actually be proven in the MIL-101-NH-[FeFe]. As catalysis ultimately leads to catalyst degradation, quantification of the loss of the CO-based IR absorption can be used to identify the sites that have been catalytically active. Such an IR investigation of MIL-101-NH-[FeFe]-high after photochemical HER reveals that *ca.* 80–85% of the [FeFe] sites within the MIL-101 were engaged in catalysis (Fig. S16, ESI†).^37^ It is interesting to note that this percentage is very similar to the maximal amount of [FeFe] sites that could be addressed by Cp_2_Co (*vide supra*). This behavior is vastly different to the situation in UiO-66-[FeFe] which shows the preservation of more than 90% of the [FeFe]-based CO-stretches in the IR spectrum after illumination experiments.^24^ This is consistent with a small portion of the [FeFe] sites in UiO-66-[FeFe] being responsible for the observed catalysis.

Further support for the accessibility of the [FeFe] sites within MIL-101-NH-[FeFe] was obtained from fluorescence microscopy measurements which revealed [Ru(bpy)_3_]^2+^ throughout the crystals, in contrast to UiO-66-[FeFe] where no emission could be detected (Fig. S17, ESI†). [Ru(bpy)_3_]^2+^ is found in MIL-101-NH-[FeFe]-high prior to as well as during photo-catalysis, supporting the notion that pore clogging may contribute to the generally low turnover rates of the materials.

In summary, we describe the spectroscopic characterization of a dianionic catalyst intermediate [FeFe]^2–^ that is stabilized in the MOF on the timescale of hours. The pore size of MIL-101 allows the chemical reduction of large proportions of the catalysts throughout the crystal, but small shifts in the IR spectrum of the reduced [FeFe]^2–^ over time suggest ion pairing phenomena with [Cp_2_Co]^+^ that limit fast and efficient catalyst accessibility. An analogous reduction experiment with UiO-66-[FeFe] produces only negligible amounts of reduced [FeFe]^2–^, consistent with the smaller pore size in this framework and pore clogging by ion pairing. Photochemical hydrogen evolution experiments using three MIL-101-NH-[FeFe] materials with different catalyst loadings show that the amount of produced hydrogen is proportional to the catalyst loading. It is further shown that a large fraction of the catalysts within the MIL-101-NH-[FeFe] engage in catalysis, as evidenced by IR spectroscopy of the materials after photocatalysis. In contrast, large proportions of catalysts in the UiO-66-[FeFe] system most likely do not participate in catalysis, as their IR spectrum after photocatalysis is to a large extent unchanged. The presented experiments show that the development of improved MOFcats will require not only careful catalyst designs, but also judicious engineering of viable transport channels through the MOF crystals.

Financial support from the Wenner-Gren Foundations (postdoctoral stipend to S. R.), the Swedish Research Council, the Swedish Energy Agency, the Berzelii Center EXSELENT on Porous Materials, the Knut & Alice Wallenberg Foundation, and the European Research Council (ERC-CoG2015-681895_MOFcat) is gratefully acknowledged. We are grateful to Dr Giannantonio Cibin for XAS measurements at beamline B18, Diamond Light Source, UK, under the Rapid Access Proposal SP14439.

## Supplementary Material

Supplementary information
