# Supplementary material for: Catalyst accessibility to chemical reductants in metal–organic frameworks
Source: Chem Commun (Camb). 2017 Feb 23;53(22):3257–60. doi: 10.1039/c7cc00022g (PMC5836565; doi:10.1039/c7cc00022g)
Supplement: Supplementary file 1 [file CC-053-C7CC00022G-s001.pdf]

Electronic Supplementary Material (ESI) for ChemComm.
This journal is © The Royal Society of Chemistry 2017

Supporting Information

Catalyst Accessibility to Chemical Reductants in Metal-Organic Frameworks

Souvik Roy,[†, a] Vlad Pascanu,[†, b] Sonja Pullen,[†, a] Greco González-Miera,[b] Belén Martín-Matute,*[,b] and Sascha Ott*[,a]

[a] Department of Chemistry, Ångström Laboratory, Uppsala University, 75120 Uppsala, Sweden
[b] Department of Organic Chemistry and Berzelii Center EXSELENT, Arrhenius Laboratory, Stockholm University, 10691 Stockholm, Sweden
[†] Authors contributed equally to this work

*Email: Prof. Dr Belén Martín-Matute (belen.martin.matute@su.se)
*Email: Prof. Dr Sascha Ott (sascha.ott@kemi.uu.se)

General methods and equipment

Reagents and solvents were used as obtained from commercial suppliers without further purification. Synthesis of MIL-101 and its post-synthetic functionalization to MIL-101-NH_2 were carried out according to previously reported procedures.[1] [$Fe_2(c_xbdt)(CO)_6$] complexes (x = 1 or 2) were synthesized using a literature procedure.[2] Powder X-Ray Diffraction (PXRD) data was collected on a PANalytical X'Pert PRO diffractometer in reflectance Bragg–Brentano geometry equipped with a pixel detector and using Cu Kα1 radiation. The incident X-ray wavelength was 1.54056 Å. Samples were dispersed on zero-background Si plates with EtOH and dried at room temperature. Thermogravimetric analyses (TGA) were performed on a Perkin Elmer TGA 7, equipped with a platinum pan. All samples were heated at a rate of 5 °C min^{-1} from 30 to 800 °C under nitrogen. N_2 sorption isotherms were measured at 77 K on a Micromeritics ASAP2020 system. MIL-101-NH_2 samples were degassed at 120 °C for 12 h prior to measurements. MIL-101-NH-[FeFe] samples were degassed at 20 °C for 12 h prior to measurements. X-ray Absorption Spectroscopy (XAS) measurements (Fe *K*-edge) were carried out at beamline B18 at Diamond Light Source, Oxford, UK. Data acquisition was performed in fluorescence mode at liquid N_2 temperature using a 36 element Ge detector. Samples were prepared as pellets. The energy scale was calibrated with an Fe foil. Data processing and analyses were performed with the Athena and Artemis software package using IFEFFIT.[3,4] The background was removed using the AUTOBK and flattening algorithm[5] implemented in Athena. The pre-edge background was linearly subtracted below the edge and a three-term polynomial function was used for the post-edge background subtraction. The pre-edge background subtracted spectra were normalized to the edge jump, E_0, which was taken as the energy of the first inflection point on the edge, i.e. the first maximum in the derivative of the absorption. The Fe K-edge Extended X-ray Absorption Fine Structure (EXAFS) reduced data χ(k) were Fourier-transformed into R-space over the range 2-11.5 $Å^{-1}$ with a k_2 weighting factor and a 3 $Å^{-1}$ Hanning window. Elemental analyses were performed by inductively coupled plasma optical emission spectrometry (ICP-OES) with a Varian Vista MPX instrument by Medac Ltd (UK). Fourier Transform Infrared (FT-IR) spectroscopy measurements for preliminary characterization of the catalysts were performed on a Varian 670-IR spectrometer equipped with an attenuated total reflection (ATR) device (Goldengate by Specac) with a single reflection diamond ATR element. IR spectra of the MOF-suspensions during reduction with $CoCp_2$ were recorded on a Perkin Elmer SpectrumOne FT-IR spectrometer using a liquid-sample-cell (Omni-Cell, Specac) equipped with CaF_2 windows and 0.5 mm PTFE spacers.

Synthesis and functionalization of the MOFs

MIL-101(Cr): $Cr(NO_3)_3·9H_2O$ (800 mg) and terephthalic acid (332 mg) were suspended together in deionized H_2O (10 mL) in a 23 mL Teflon-lined steel autoclave. HF (1 mL, 2M aqueous solution) was added dropwise, the autoclave was sealed, sonicated for 15 min and heated at 220 °C for 16 h in an oven. After cooling to room temperature, the impurities were filtered-off through a sintered funnel (P2). The filtrate was centrifuged (9000 rpm, 10 min), and the solvent was decanted, affording small dark green crystals. The MOF crystals obtained after decantation of the supernatant were further activated as described in the literature, in order to remove impurities form the pores.[1a]

MIL-101-NO_2: H_2SO_4 (105 mL, conc.) was added slowly over HNO_3 (75 mL, conc.) in a flask cooled on an ice/NaCl bath and the acid mixture was stirred for 10 min. MIL-101 (1.5 g) was added portion-wise over 30 min and the suspension was further stirred for 6 h while maintaining the temperature between -10 °C and -5 °C. The mixture was then poured over ice (500 mL) and further diluted with deionized H_2O (500 mL). After the ice melted, the suspension was centrifuged at low temperature (9000 rpm, 10 min, 4 °C). The MOF crystals were recovered by decantation and washed several times with cold H_2O to remove acid traces. For further clearing the pores, the MOF was finally stirred in hot

H_2O (100 mL, 90 °C, 1 h) and hot EtOH (100 mL, 70 °C, 1 h). The activated MOF was recovered upon centrifugation, dried under vacuum and subjected to the reduction reaction.

MIL-101-NH$_2$: MIL-101-NO$_2$ (1350 mg) was suspended in EtOH (99.5%, 240 mL) and heated to 70 °C. $SnCl_2 \cdot 2H_2O$ (45 g) was added portion-wise and the mixture was further stirred at constant temperature for 6 h. After cooling down, the MOF was recovered by centrifugation, suspended in HCl (100 mL, conc.) and stirred for 1 h at room temperature to wash away Sn residues from the pores. Further activation steps were performed as described in the literature.[1c]

MIL-101-NH-[FeFe]-*high* (5.28 wt% Fe): Anhydrous CH_2Cl_2 was collected from a VAC© solvent purification column, subjected to 3 freeze-pump-thaw cycles and stored over MS (4 Å). $Fe_2(c_1bdt)(CO)_6$ (35 mg, 0.075 mmol) and Et_3N (17 µL, ~0.12 mmol) were dissolved in CH_2Cl_2 (20 mL) under Ar and the solution was cooled to 0 °C, in a flask covered in aluminium foil. Ethyl chloroformate (10 µL, ~0.1 mmol) dissolved in CH_2Cl_2 (5 mL) was added dropwise to the first solution. The mixture was further stirred for 20 min at 0 °C. Subsequently, the solution was transferred using a cannula to a separate flask covered in aluminium foil containing MIL-101-NH$_2$ (50 mg) under Ar. Prior to this, the MOF-containing flask had been kept under vacuum at 100 °C for 12 h, to remove all solvent molecules from the MOF's pores. After which the flask was carefully degassed and filled with argon. After transferring the Fe complex, the suspension was stirred at room temperature for 72 h. The suspension was then transferred to a Falcon tube trying to minimize exposure to air. Centrifugation was performed at 9000 rpm for 10 min, in a pre-cooled centrifuge at 4 °C. The supernatant had a pale orange color, suggesting the impregnation of Fe was not quantitative. The resulting solid was subjected to three washing cycles with anhydrous CH_2Cl_2 (3 x 50 mL), under Ar (the Falcon tube's cap was replaced with a septum, flushed with Ar and shaken for 30 min. at 50 rpm, followed by centrifugation). After 3 cycles the supernatant became completely colorless. The resulting functionalized MOF was dried under vacuum for 12 h at room temperature, avoiding exposure to light.

MIL-101-NH-[FeFe]-*medium* (2.35 wt% Fe) was synthesized by a similar procedure using MIL-101-NH$_2$ (50 mg), $Fe_2(c_1bdt)(CO)_6$ (15 mg, 0.032 mmol), Et_3N (8 µL) and ethyl chloroformate (5 µL). The time allowed for impregnation was 36 h.

MIL-101-NH-[FeFe]-*low* (0.59 wt% Fe) was synthesized by a similar procedure using MIL-101-NH$_2$ (50 mg), $Fe_2(c_1bdt)(CO)_6$ (10 mg, 0.0215 mmol), Et_3N (5 µL) and ethyl chloroformate (3 µL). The time allowed for impregnation was 16 h.

Chemical reduction experiments: All samples were prepared inside a dry Ar-glovebox. A dispersion of MOF material (4 mg mL^{-1}) was prepared by sonicating the respective MOF in acetonitrile under Ar for 30 min. A stock solution of $CoCp_2$ (25 mM) in acetonitrile was prepared separately. Different aliquots of $CoCp_2$ solution were added to 0.25 mL MOF-suspension to achieve the following concentration ratios, [$CoCp_2$]:[Fe_2] = 5, 10, 20 and 40. The resulting mixtures were diluted to 0.4 mL to give a 2.5 mg mL^{-1} MOF suspension. FTIR spectra of the suspensions were measured within 5-10 min of starting the reaction. For the time dependent studies, an excess of cobaltocene (10 equivalents with respect to the amount of catalyst present) was added to the suspension of MOF (~1.5 mg mL^{-1} in acetonitrile) and FTIR spectra were recorded at regular intervals (5, 20, 50, 110, 180 and 240 min).

Reduction of $Fe_2(bdt)(CO)_6$: An acetonitrile solution of $Fe_2(bdt)(CO)_6$ (9 mg, 0.021 mmol, 1 mL) was mixed with a cobaltocene solution (41 mg, 21.3 mmol, 3 mL MeCN) under inert atmosphere (glovebox) and IR spectrum of the solution was recorded at regular intervals.

Photocatalysis: An aqueous dispersion of MOF (0.75 mg mL^{-1}) was prepared by sonicating the MOF in 1 M acetate buffer. A stock solution containing $Ru(bpy)_3Cl_2$ (1 mM) and sodium ascorbate (200 mM) in 1 M acetate buffer was prepared separately and the pH was adjusted to 4.9. This solution (2 mL) was added to the MOF-dispersion (2 mL) and the reaction vessel was sealed (total volume = 4 mL, MOF = 1.5 mg, [$Ru(bpy)_3Cl_2$] = 0.5 mM, [ascorbate] = 100 mM). After sparging with Ar for 30 min, the mixture was irradiated with blue LED (470 nm) for 6-7 hours. The headspace was sampled by GC to quantify the H_2 evolved. Prior to each experiment, the GC was calibrated using 7.5 % H_2 in argon gas mixture. After photocatalysis, the MOF-material was collected by centrifugation, thoroughly washed with water, and dried under vacuum. The dry MOF was re-dispersed in acetonitrile (0.5 mL) and the FTIR spectrum was recorded (Fig. S9). Note: Approximately 10-20% (wt) MOF was lost during isolation of the material after photocatalysis experiments.

To record fluorescence microscope images, the photocatalysis was stopped after 30 min. Then the MOF-material was collected by centrifugation, quickly washed with water (1 mL) and methanol (1 mL), and finally dried under vacuum. The fluorescence microscope images were recorded using λ_{ex} = 478 nm and λ_{em} = 550 – 642 nm.

Calculation of number of moles of catalyst inside MOF

Molecular formula for MIL-101-Fe_2 is $(Cr_3F)(H_2O)_2O[(BDC\text{-}NH_2)_{3-x}(BDC\text{-}NH\text{-}Fe_2)_x].nH_2O$; (n = 25 and x = number of catalyst-functionalized linkers)[1a]

Table S1. Elemental analyses of MIL-101-NH-[FeFe] with different catalyst loadings

Element (wt%)	**C**	**H**	**N**	**S**	**Fe**
High loading	36.88	3.54	3.99	4.33	**5.28**
Medium loading	37.92	4.58	3.71	3.72	**2.35**
Low loading	34.29	3.80	4.31	1.26	**0.59**

Table S2. Catalyst concentration inside the MOF; x = number of catalyst-functionalized linkers; $n_{cat.}$/mg MOF = µmol of catalyst in 1 mg MOF material

MOF	% Fe	x	MW_{MOF}	$n_{cat.}$/mg MOF
MIL-101-NH-[FeFe]-*high*	5.28	0.727	1540.46	0.192
MIL-101-NH-[FeFe]-*medium*	2.35	0.282	1340.67	0.098
MIL-101-NH-[FeFe]-*low*	0.59	0.066	1244.34	0.027

Turnover numbers (TON) under photocatalytic condition

TON is defined as the number of moles of H_2 produced by one mole of catalyst. However, it is important to note that, in the present case where a molecular catalyst-MOF hybrid material is used, this TON is the lower estimate as some deeply buried catalytic sites might not be accessible during catalysis.

Table S3. Overall turnover numbers (TON) of the MOFcats under photocatalytic conditions

Time (min)	TON		
	MIL-101-NH-[FeFe]-*high*	MIL-101-NH-[FeFe]-*medium*	MIL-101-NH-[FeFe]-*low*
35	4.5	7	5.1
72	9.7	11	8.6
100	14	14.1	11.4
140	16.8	18.5	13.3
360	22.3	26.5	18.3

Reduction of MIL-101 bound [FeFe] catalysts by cobaltocene

The degree of conversion from [FeFe] to $[FeFe]^{2-}$ was determined from the intensity of the C-O stretching bands in the FTIR spectra of the MOF suspensions. The decay of the [FeFe] and the growth of $[FeFe]^{2-}$ was monitored form the absorbance of ν_{C-O} bands at 2076 and 1882 cm^{-1}, respectively. The yields of the reduction reaction listed below were determined after 10 min.

Table S4. Relative amount of reduced [FeFe]-catalyst inside MOF upon incremental addition of Cp_2Co

[Cp_2Co, µmol]/[FeFe, µmol]	% reduced catalyst (±5)	
	MIL-101-NH-[FeFe]-*high*	MIL-101-NH-[FeFe]-*medium*
5	36	54
10	55	69
20	71	78
40	82	90

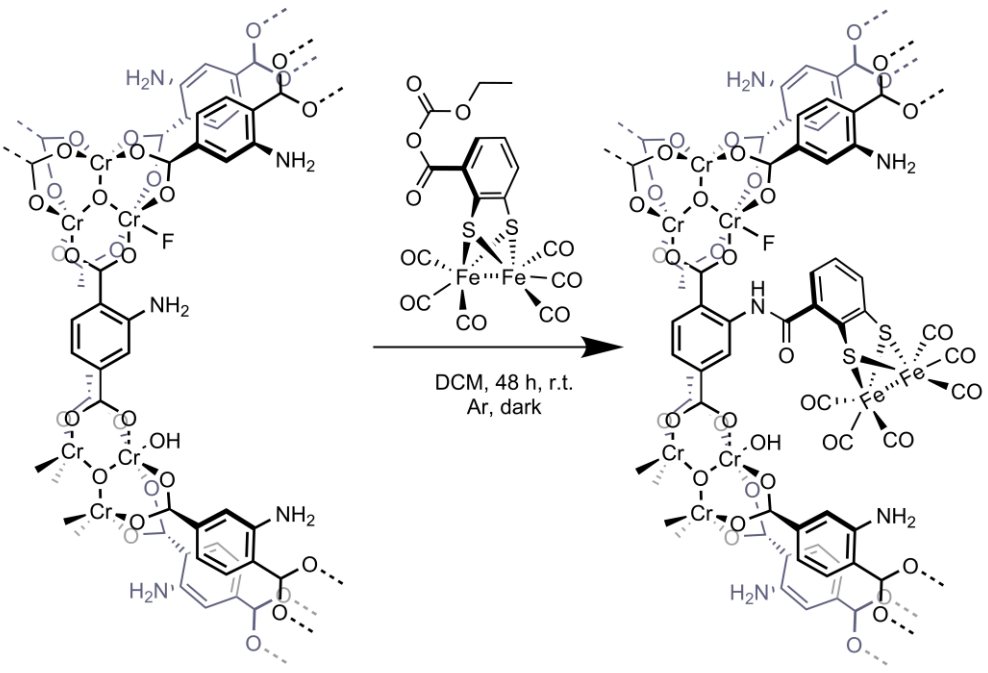

MIL-101-NH$_2$ MIL-101-NH-[FeFe]

Scheme S1. Preparation of MIL-101(Cr)-NH-[FeFe] from a reaction between MIL-101(Cr)-NH$_2$ and a mixed anhydride of the [$Fe_2(c_1bdt)(CO)_6$] ([FeFe] catalyst).

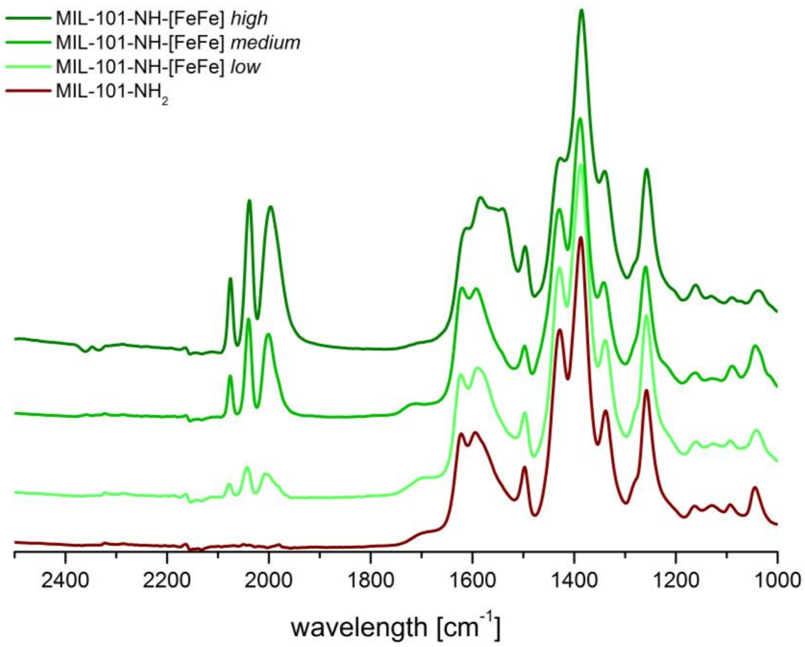

Figure S1. FTIR spectra of MIL-101-NH$_2$ before and after functionalization with various amounts of $Fe_2(c_1bdt)(CO)_6$.The IR spectrum of MIL-101-NH-[FeFe]-*high* shows an additional band at 1537 cm^{-1}. This is also visible in the parent MOF and does not come from the functionalization process. MIL-101-NH-[FeFe]-*high* was synthesized from a different MOF batch than the other two. This extra band in IR fits corresponds to the N–O asymmetric stretch of MIL-101-NO$_2$ and appears due to incomplete reduction of -NO$_2$ to -NH$_2$ groups in this batch. This inconsistency did not affect the MOF functionalization process or the catalytic properties of the material.

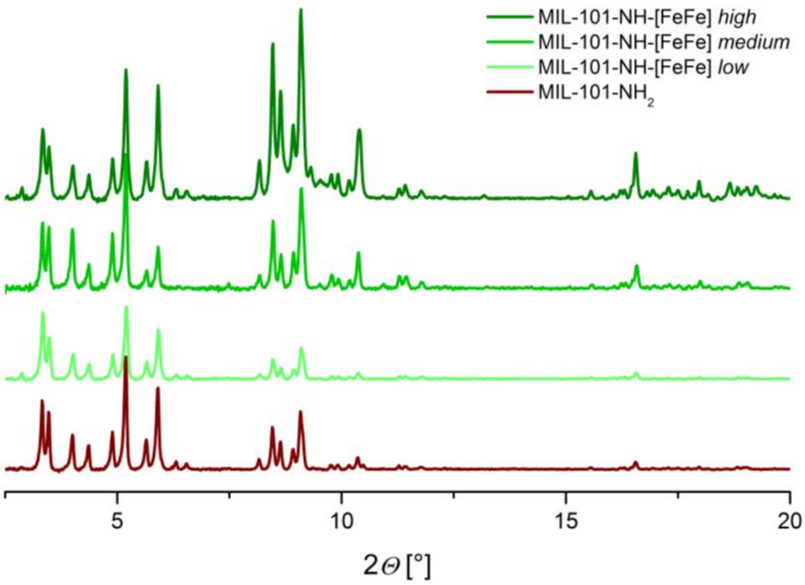

Figure S2. PXRD patterns of MIL-101-NH$_2$ before and after functionalization with $Fe_2(c_1bdt)(CO)_6$.

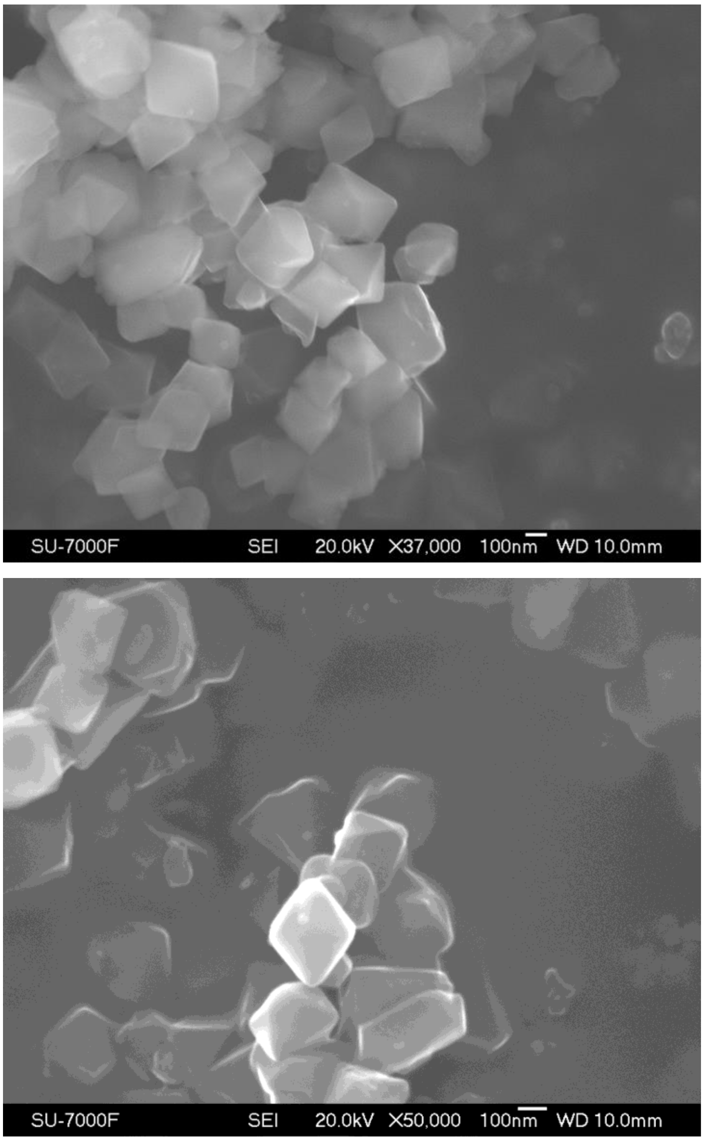

Figure S3. SEM images of MIL-101-NH-[FeFe]-*high*

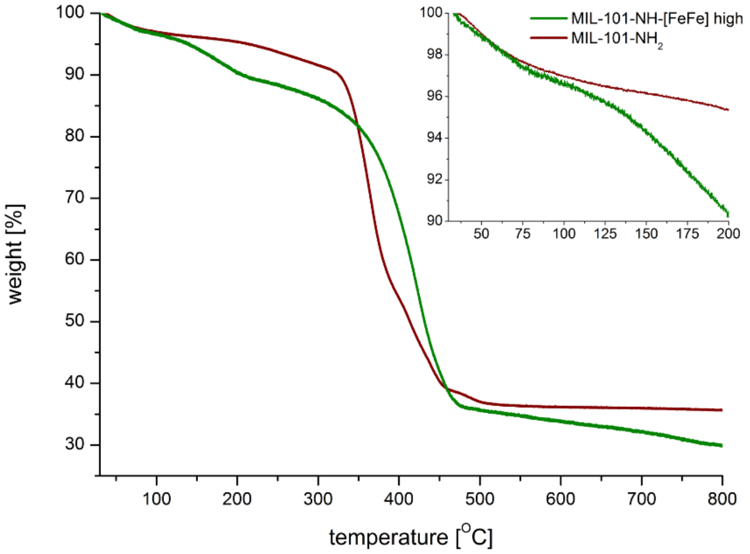

Figure S4. TGA profiles of MIL-101-NH_2 before and after functionalization with $Fe_2(c_1bdt)(CO)_6$ (high catalyst loading). The TGA of MIL-101-NH_2 shows the expected gradual loss of guest molecules until decomposition of the organic linkers at *ca*. 365 °C. The functionalized MOFs show an additional step at low temperature (90 – 230 °C), matching the decomposition profile of similar metal-carbonyl complexes (inset).[2,6] For MIL-101-NH-[FeFe]-*high*, this corresponds to 7% weight losses.

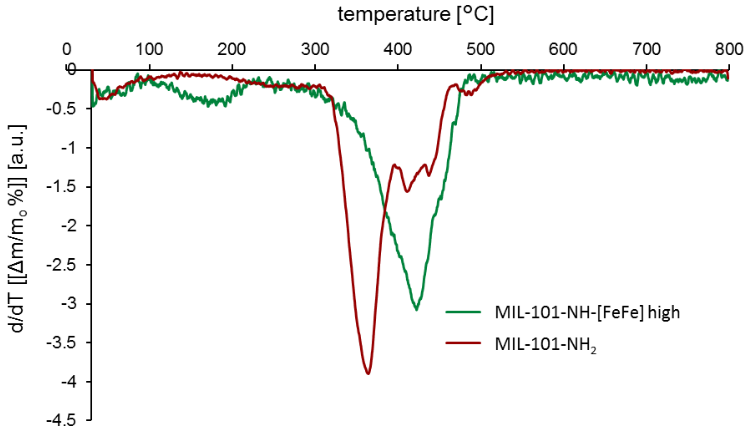

Figure S5. First derivative of TGA profiles (dTGA) of MIL-101-NH_2 before and after functionalization with $Fe_2(c_1bdt)(CO)_6$ (high catalyst loading).

BET Surface Area Measurement. N_2 sorption isotherms were recorded and BET surface areas calculated for the materials with *high* and *low* catalyst loadings. For **MIL-101-NH-[FeFe]** ***high***, a drop of **38%** in surface area was obtained compared to its corresponding batch of unfunctionalized MOF. For **MIL-101-NH-[FeFe]** ***low***, a drop of **28%** in surface area was obtained compared to its corresponding batch of unfunctionalized MOF.

However, the interpretation of these results should be done with caution. For this procedure, MOFs are typically activated under vacuum for 12 h at 120 °C to remove all volatile guest molecules. It is clear from TGA that such a treatment also decomposes the catalyst. Therefore, catalyst-loaded MOF batches were activated for the same amount of time, under vacuum at ambient temperature. It

may be possible that the evacuation of guest molecules was not quantitative in these cases. Calculated surface area differences would then be unreliable because the unwanted guests will also contribute to the drop in the BET value.

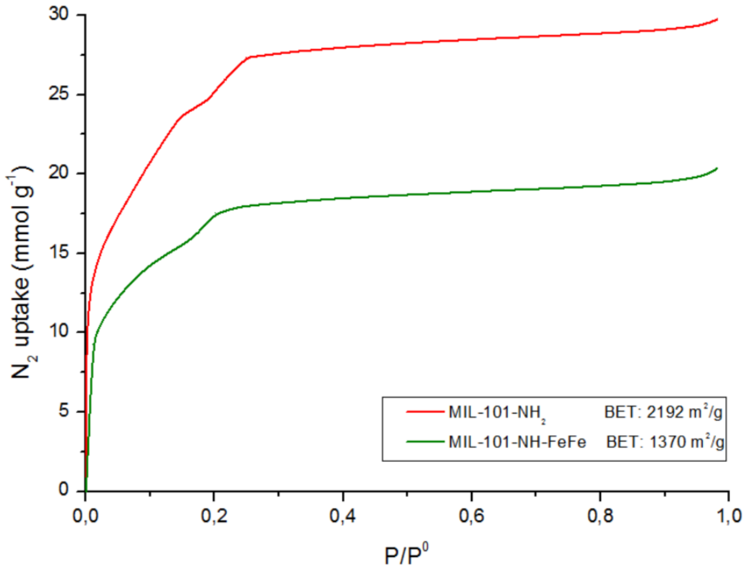

Figure S6. N_2 sorption isotherms of MIL-101-NH_2-[FeFe]-high before and after functionalization with $Fe_2(cbdt)(CO)_6$.

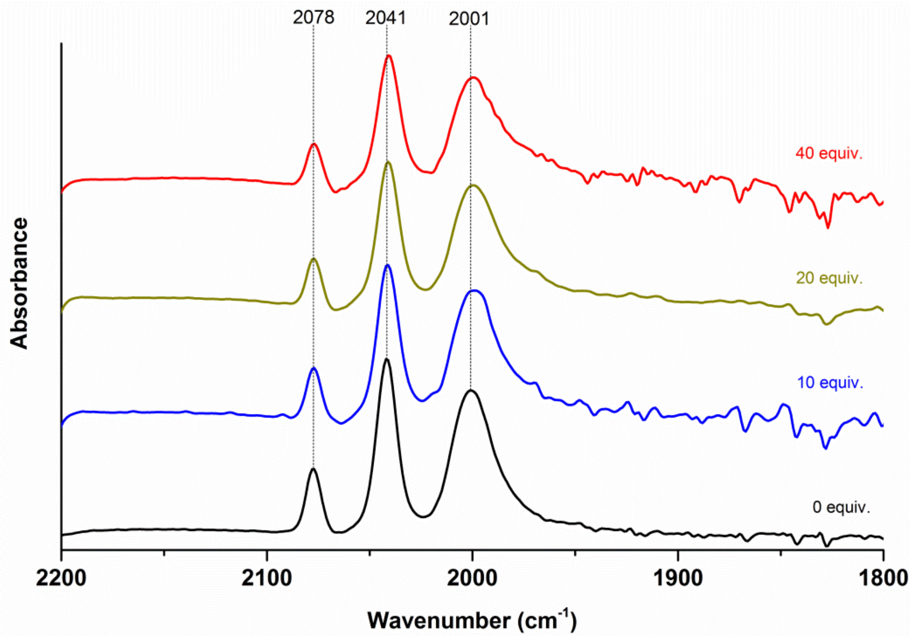

Figure S7. FTIR spectra of UIO-66-[FeFe] (1.8 mg mL^{-1} in CH_3CN, ~0.21 mM catalyst) in the presence of different amounts of cobaltocene (0–40 equiv. with respect to the amount of catalyst). For MOF synthesis, see ref. 2.

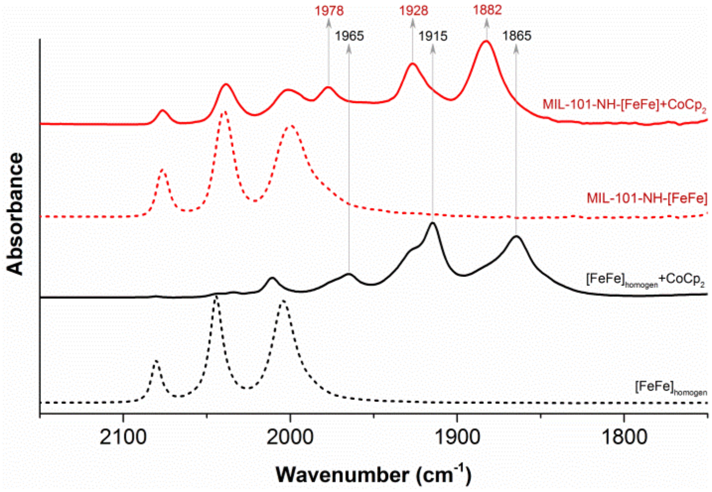

Figure S8. FTIR-spectral of a homogeneous acetonitrile solution of $Fe_2(bdt)(CO)_6$ (black) and a suspension of MIL-101-NH-[FeFe]-*medium* (red) upon addition of cobaltocene. Conditions: molar ratio, [FeFe-catalyst]:[$CoCp_2$] = 1:10; spectra recorded after 5 min of adding $CoCp_2$.

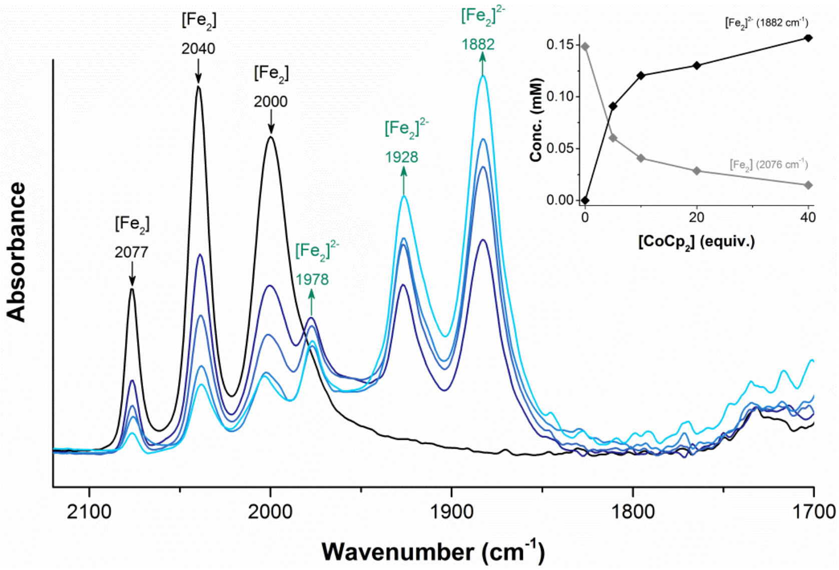

Figure S9. FTIR spectra of MIL-101-NH-[FeFe]-*medium* (1.5 mg mL^{-1} in CH_3CN, ~0.15 mM catalyst) in the presence of different amounts of $CoCp_2$ (5, 10, 20, and 40 equiv. with respect to the amount of catalyst). The black trace shows the spectrum before addition of $CoCp_2$. Inset: change in concentration of [Fe_2] and [Fe_2]$^{2-}$ with increasing amount of $CoCp_2$.

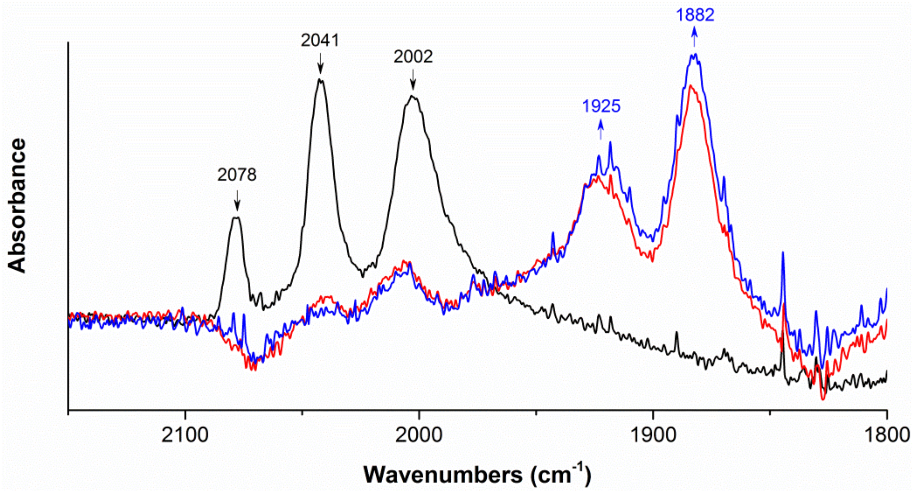

Figure S10. FTIR spectra of MIL-101-NH-[FeFe]-*low* (1.5 mg mL^{-1} in CH_3CN, ~0.04 mM catalyst) in the presence of different amounts of $CoCp_2$ (0 equiv. black; 20 equiv. red; 40 equiv. blue).

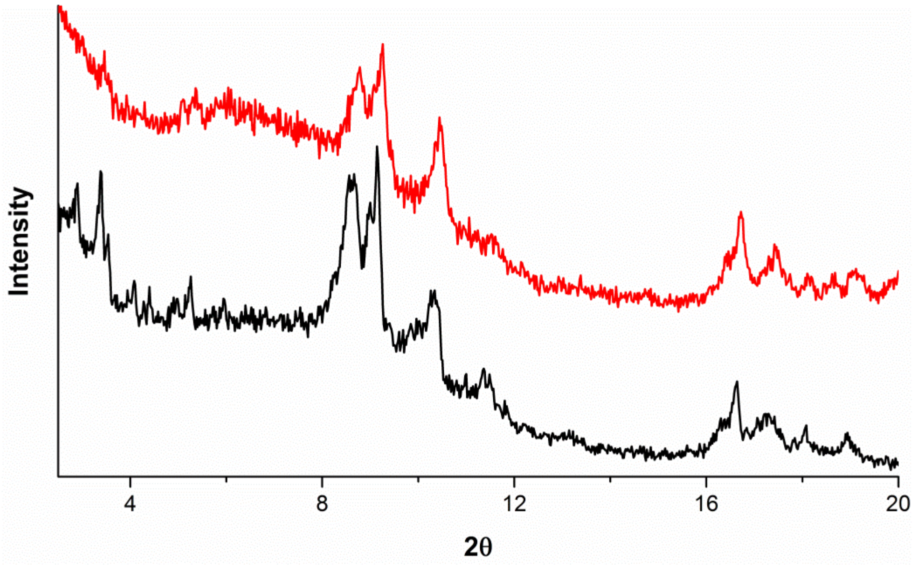

Figure S11. PXRD patterns of MIL-101-NH_2-[FeFe]-*high* before (black) and after (red) overnight treatment with 20 equiv. $CoCp_2$.

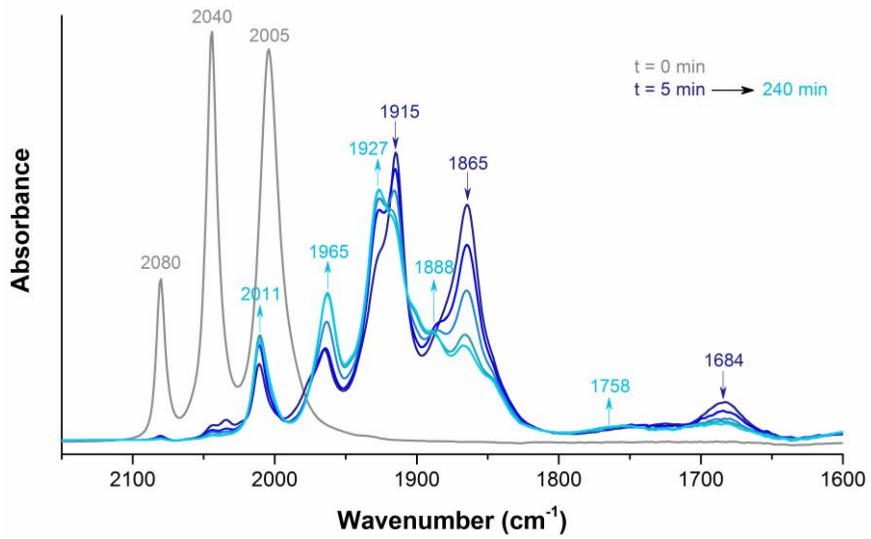

Figure S12. Time dependent FTIR-spectral changes of an acetonitrile solution of $Fe_2(bdt)(CO)_6$ (5.4 mM) and cobaltocene (54 mM). The grey trace shows the FTIR spectrum of $Fe_2(bdt)(CO)_6$ (5.4 mM in CH_3CN) before adding cobaltocene. Following the addition of $CoCp_2$, the spectra were recorded after 5, 30, 90, 180, and 240 minutes.

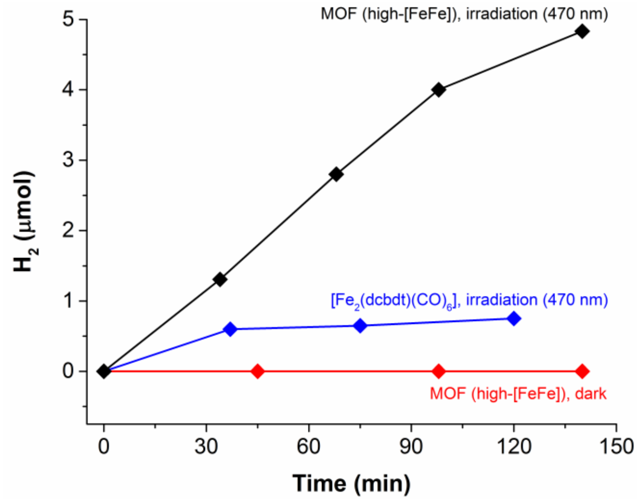

Figure S13. Photocatalytic H_2 evolution by the MOF-catalyst, MIL-101-NH-[FeFe]-*high* (black, ~0.29 µmol cat.), and by the molecular catalyst in solution, $Fe_2(dcbdt)(CO)_6$ (blue trace, 0.29 µmol). The red trace shows the control experiment for MOF-catalyst in dark and it demonstrates that the MOF-catalyst is inactive in the absence of light. Condition: 1.5 mg MIL-101-NH-[FeFe]-*high* or 0.13 mg $Fe_2(dcbdt)(CO)_6$ in 4 mL 1 M acetate buffer (pH 4.9) in the presence of 0.5 mM $Ru(bpy)_3Cl_2$ and 0.1 M ascorbic acid.

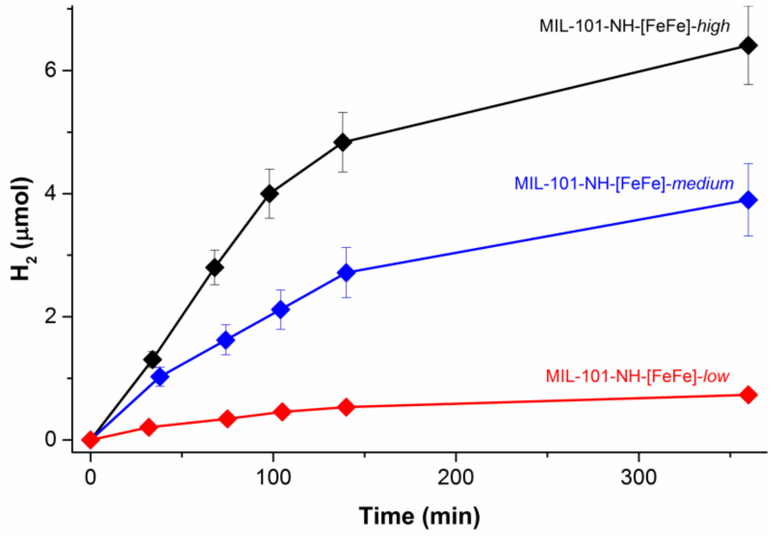

Figure S14. Photocatalytic hydrogen production in the presence of heterogeneous MOF-catalysts: MIL-101-NH-[FeFe]-*high* (red), -*medium* (green), and -*low* (blue). Reaction conditions: MOF (1.5 mg), ascorbic acid (0.1 M), $Ru(bpy)_3Cl_2$ (5×10^{-4} M), pH 5 (1.0 M acetate buffer solution, 4 mL).

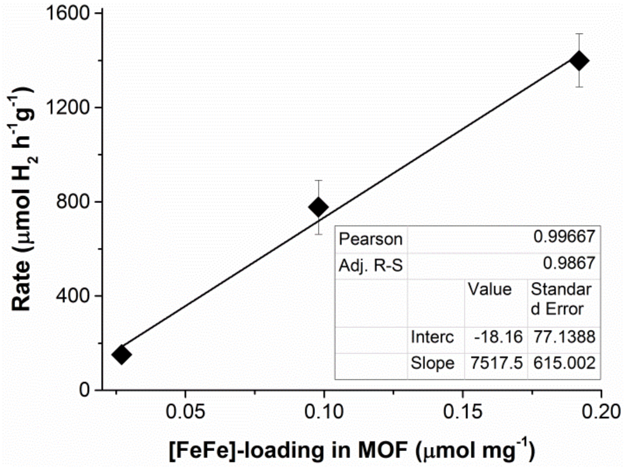

Figure S15. Dependence of the initial average H_2 evolution rate on the catalyst loading. The rate was determined from the amount of H_2 produced during the initial 2.5 h photolysis.

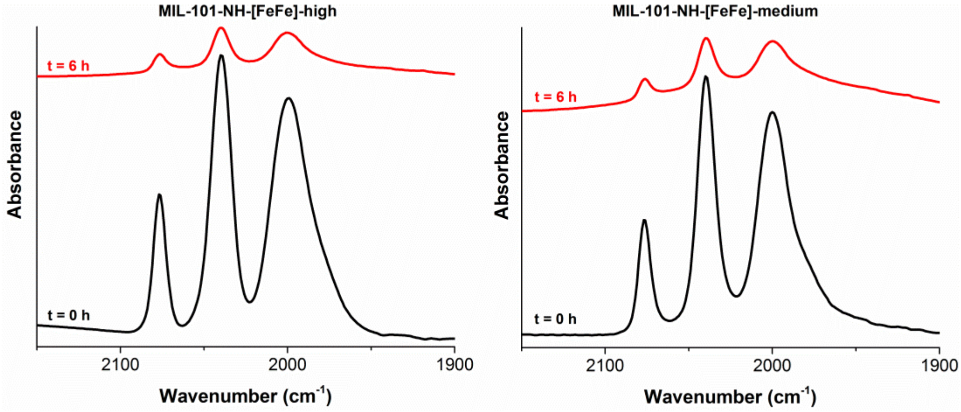

Figure S16. FTIR spectra of MIL-101-NH-[FeFe]-*high* and MIL-101-NH-[FeFe]-*medium* before (black) and after (red) light-driven hydrogen production for 6 hours. From the difference in absorbance of the CO stretching band at 2076 cm^{-1}, it can be estimated that 85(±5)% and 80(±5)% catalyst decomposed for MIL-101-NH-[FeFe]-*high* and -*medium*, over the course of photocatalysis.

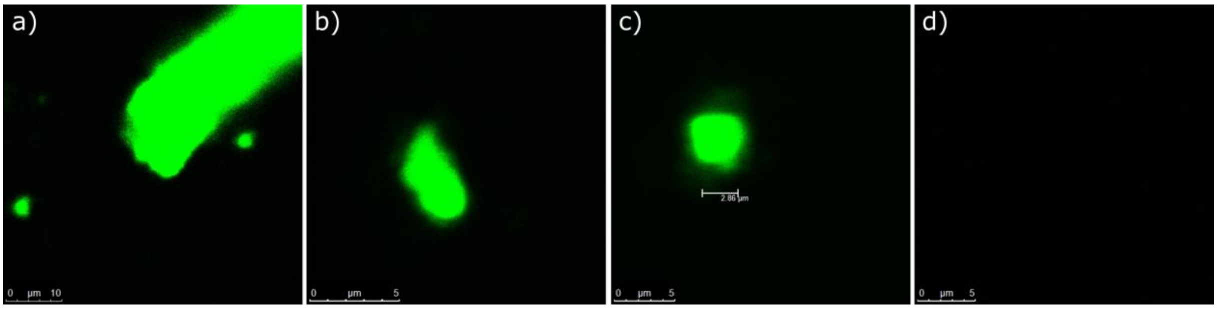

Figure S17. Fluorescence microscope images: (a) MIL-101-NH-[FeFe]-*high* (~0.375 mg mL^{-1}) soaked in a 0.5 mM $[Ru(bpy)]^{2+}$ solution for 2 h; (b, c) MIL-101-NH-[FeFe]-*high* isolated after 30 min photocatalysis (samples from two photocatalysis experiments are shown); (d) UiO-66-[FeFe] isolated after 30 min photocatalysis. The MOF crystals were excited with λ_{ex} = 478 nm and the emission in the range 550–642 nm was recorded. Prior to recording fluorescence microscope images, all MOFs were briefly washed with water (1 mL) and methanol (1 mL), and dried under vacuum.

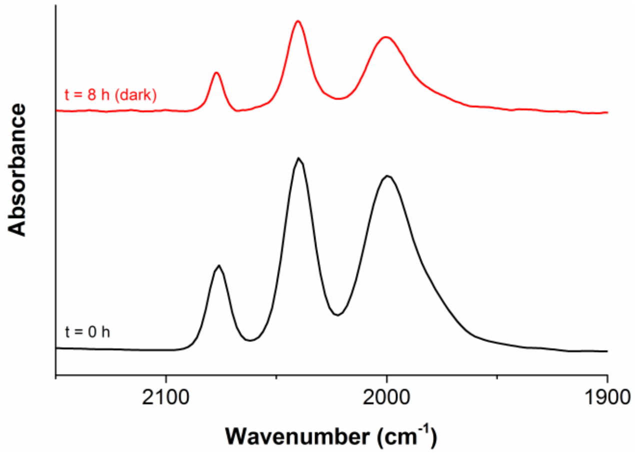

Figure S18. FTIR spectra of MIL-101-NH-[FeFe]-*high* recorded in dark in the presence of all components for photocatalysis (photosensitizer, electron donor and catalyst in acidic buffer) to probe the stability of the catalyst. The black trace shows the initial spectrum of the MOF suspension and the red trace shows the spectrum after stirring the suspension in dark for 8 h. No hydrogen was detected over the course of 8 h. The loss of intensity of the CO stretching band at 2076 cm^{-1}

corresponds to decomposition of ca. 40(±10)% catalyst over 8 h. Conditions: 1.5 mg MOF, 0.5 mM $Ru(bpy)_3Cl_2$, 0.1 M ascorbic acid, 1 M acetate buffer (pH 4.9, 4 mL).

---

[1] For MOF synthesis see: a) G. Férey, C. Mellot-Draznieks, C. Serre, F. Millange, J. Dutour, S. Surblé and I. Margiolaki, *Science*, **2005**, 309, 2040 – 2042. Alternatively, see: b) V. Pascanu, Q. Yao, A. Bermejo Gómez, M. Gustafsson, Y. Yun, W. Wan, L. Samain, X. Zou and B. Martín-Matute, *Chem. Eur. J.*, **2013**, 19, 17483 – 17493. For post-synthetic functionalization with NH_2, see: c) S. Bernt, V. Guillerm, C. Serre and N. Stock, *Chem. Commun*., **2011**, 47, 2838 – 2840.

[2] S. Pullen, H. Fei, A. Orthaber, S. M. Cohen, S. Ott, *J. Am. Chem. Soc.*, **2013**, 135, 16997–17003

[3] M. Newville, *J. Synchrotron Rad.* **2001,** 8, 322 – 324.

[4] B. Ravel, M. Newville, *J. Synchrotron Rad.* **2005**, 12, 537 – 541.

[5] M. Newville, P. Livins, Y. Yacoby, E. Stern, J. Rehr, *Phys. Rev. B: Condens. Matter Mater. Phys.* **1993**, 47, 14126 – 14131.

[6] H. Fei, M. D. Sampson, Y. Lee, C. P. Kubiak, S. M Cohen, *Inorg. Chem.* **2015**, *54*, 6821 – 6828.
